# Comparison of Electrocoagulation and Conventional Medical Drops for Treatment of Conjunctivochalasis: Short-Term Results

**DOI:** 10.4274/tjo.35002

**Published:** 2018-04-25

**Authors:** Mehtap Çağlayan, Pınar Kösekahya, Canan Gürdal, Özge Saraç

**Affiliations:** 1 Mardin State Hospital, Ophthalmology Clinic, Mardin, Turkey; 2 Ulucanlar Ophthalmology Training and Research Hospital, Ophthalmology Clinic, Ankara, Turkey; 3 Bozok University Faculty of Medicine, Department of Ophthalmology, Yozgat, Turkey; 4 Atatürk Training and Research Hospital, Ophthalmology Clinic, Ankara, Turkey

**Keywords:** Conjunctiva, electrocoagulation, optical coherence tomography

## Abstract

**Objectives:**

To compare the effectiveness of electrocoagulation and conventional medical drops for treatment of conjunctivochalasis using anterior segment-optical coherence tomography (AS-OCT).

**Materials and Methods:**

Forty eyes of 20 patients with bilateral conjunctivochalasis were included in this prospective study. Twenty eyes of 10 patients were assigned to Group 1 and underwent electrocoagulation. The other 20 eyes of 10 patients were assigned to Group 2 and received conventional medical treatment consisting of non-steroidal antiinflammatory drop (topical 0.5% ketorolac tromethamine) 4 times a day and artificial tears (0.15% sodium hyaluronate) 6 times a day for 4 weeks. Before and 4 weeks after treatment, all patients were evaluated by slit-lamp biomicroscopy, tear film break-up time (TBUT) test, and ocular surface disease index (OSDI) questionnaire. Tear meniscus height (TMH), tear meniscus area (TMA), and conjunctivochalasis area (CCA) were measured with AS-OCT.

**Results:**

In Group 1, posttreatment values of TMH, TMA, and TBUT were significantly higher (p<0.001, p=0.006, and p<0.001, respectively), while CCA and OSDI scores were significantly lower than pretreatment values (p<0.001 for both values). In Group 2, only OSDI decreased significantly between pretreatment and posttreatment values (p<0.001). The other parameters did not change significantly after treatment (p>0.05 for all values).

**Conclusion:**

Electrocoagulation is an effective modality for treatment of conjunctivochalasis

## Introduction

Conjunctivochalasis (CCh) was first described at 1942 by Hughes^1^ as redundant, loose, nonedematous inferior bulbar conjunctiva interposed between the globe and the lower eyelid. Increased collagenolytic activity and conjunctival elastic fibril degeneration related to oxidative stress, and ocular surface inflammation are responsible for the etiopathogenesis of CCh, but neither is solely responsible for the disease.^1,2,3,4^ CCh is a clinical diagnosis but is generally overlooked by clinicians. Patients with CCh can be asymptomatic or symptomatic. Symptoms can vary along a broad spectrum, ranging from tearing, itching, and eye burning to gritty feeling, localized pain, ulceration, and subconjunctival hemorrhage. Asymptomatic cases do not require treatment, while symptomatic cases usually require medical and/or surgical treatment. Artificial tears are commonly used in CCh treatment due to tear film instability and dry eye symptoms in eyes with CCh.^5^ In addition, topical anti-inflammatory eye drops are often prescribed in clinical practice to reduce ocular surface inflammation.^5^ The most preferred surgical technique is crescent-shaped conjunctival excision and primary suturation.^5^ Other surgical procedures include amniotic membrane transplantation with fibrin glue and suturing the CCh tissue directly to sclera.^5,6,7^ Electrocauterization of loose conjunctival tissue is another non-surgical modality for the treatment of CCh. It is applied 5 mm from the limbus and causes no damage to the fornices.^8,9,10^

Anterior segment-optical coherence tomography (AS-OCT) has rapidly become a valuable tool in the field of ophthalmology. Dry eye evaluation by measuring the tear meniscus and tear film with AS-OCT has been well described previously.^11,12,13,14^ AS-OCT has also been described as a useful and reproducible instrument to measure the cross-sectional area of conjunctiva prolapsing into the tear meniscus of eyes with CCh.^8^

In this study, we aimed to use AS-OCT to compare the effectiveness of electrocoagulation and conventional medical drops in the treatment of CCh.

## Materials and Methods

This prospective study was conducted in compliance with the institutional and government review board regulations, informed consent regulations, and the Declaration of Helsinki. Written informed consent, which was approved by our institutional ethics committee (approvel number: 26379996/122), was obtained from all patients.

The study included 40 eyes of 20 consecutive patients with CCh suffering from dry eye symptoms. Twenty eyes of 10 patients were treated with electrocauterization (Group 1), and the other 20 eyes of 10 patients were treated with conventional medical drops including non-steroidal antiinflammatory agent (topical 0.5% ketorolac tromethamine) and artificial tears (0.15% sodium hyaluronate) (Group 2). Patients who wore contact lenses, had any ocular surface disease or eyelid disorder, history of previous eye surgery, or systemic and/or topical medication usage within the last year, as well as any patients who were pregnancy or breastfeeding were excluded from the study.

All patients were evaluated with the Ocular Surface Disease Index questionnaire (OSDI), tear film break-up time (TBUT) measurement, and a complete ophthalmologic examination including visual acuity measurement, slit-lamp biomicroscopic anterior and posterior segment examination, and intraocular pressure measurement. CCh was diagnosed biomicroscopically as redundant, loose conjunctival tissue between the inferior eyelid margin and globe. CCh was graded according to Lid Parallel Conjunctival Folds (LIPCOF) grading system, using the method described by Höh et al.^15^ (Table 1).

Ocular symptoms were analyzed with the OSDI, a 12-item questionnaire consisting of 3 categories (ocular symptoms, environmental factors, and visual functions).^16^ Each OSDI item is scored on a scale from 0 (never) to 4 (always). Total OSDI score was calculated with the following formula: Total OSDI score= (Sum of scores *25)/number of questions answered.^16^ The overall OSDI scores range from 0 to 100.

Fourier-domain OCT (RTVue, software version 2.7; Optovue Inc., Fremont, California, USA) anterior segment module (Cam-L) was used for measuring tear meniscus parameters including tear meniscus area (TMA), tear meniscus height (TMH), and CCh area (CCA). All measurements were made by the same clinician (M.C.). The patient was seated in front of the OCT device, asked to look straight ahead after blinking and the inferior temporal region was scanned in the vertical plane in 2 seconds. Unlike previous studies, the CCA, TMH, and TMA values were measured manually from the inferotemporal wedge region between the bulbar conjunctiva and eyelid margin.

After AS-OCT measurement, TBUT was evaluated. Fluorescein 2% solution was instilled into the inferior fornix before asking the patient to blink three times and then look straight forward. The tear film was analyzed by slit-lamp biomicroscopy with cobalt blue filter. The elapsed time until initial break-up, rupture of the tear film, or formation of any tiny dry spots was recorded. TBUT was measured three times and the measurements were averaged.^17^

Electrocauterization was performed on the eyes in Group 1 according to the method described below. After the instillation of topical anesthetic, the patients were seated in front of the slit-lamp biomicroscope and instructed to look upward. In order not to cause any corneal injury, the excess conjunctiva was grasped 5 mm away from the limbus at the inferotemporal region and coagulated with low-voltage manual electrocautery. Coagulation was considered to be adequate when the conjunctiva turned white. Coagulation was performed at approximately 10 sites in an arc on the inferior temporal bulbar conjunctiva. Postoperatively, topical antibiotic drops (0.3% lomefloxacin) were prescribed 4 times daily and stopped 1 week later.

Conventional medical drops were prescribed to the eyes in Group 2. Non-steroidal antiinflammatory drops (topical 0.5% ketorolac tromethamine) 4 times a day and artificial tears (0.15% sodium hyaluronate) 6 times a day were administered for 1 month. The patients in the second group were monitored for treatment compliance and the patients who could not adhere to the treatment were excluded from the study. All medications were discontinued 3 days before the post-treatment examinations. All examinations were performed in both groups before and 4 weeks after treatment.

### Statistical Analysis

Statistical analysis was performed with SPSS 17.0 for Windows (SPSS Inc, Chicago, Illinois, USA). Normality of data was analyzed with a Shapiro-Wilk test. Descriptive statistics were performed as mean ± standard deviation. Statistical analyses were performed using independent samples t-test and paired samples t-test. Categorical variables between the groups were analyzed using chi-square (x^2^) tests. A p value of less than 0.05 was considered statistically significant.

## Results

The mean age was 57.6±9.9 years in Group 1 and 59.2±8.10 years in Group 2 (p=0.29). There were 5 (50%) females and 5 (50%) males in Group 1 and 7 (70%) females and 3 (30%) males in Group 2 (p=0.36).

The LIPCOF grading (Table 1) distribution was similar between groups: 8 eyes were in grade 2 and 12 eyes were in grade 3 in Group 1, while 9 eyes were in grade 2 and 11 eyes were in grade 3 in Group 2 (p=0.74).

**Figure 1:**
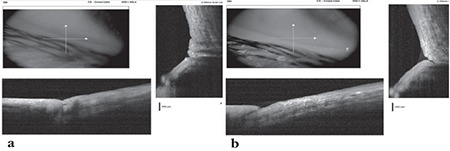
Optical coherence tomography view before (a) and after (b) electrocauterization

In Group 1, the mean TMA and TMH values significantly increased after treatment (p=0.006 and p<0.001 respectively). CCA decreased from 0.35±0.18 to 0.10±0.07 mm^2^ after treatment (p<0.001) (Figure 1). Mean TBUT increased and OSDI score decreased after treatment (p<0.001 and p<0.001) (Table 2), indicating improvement of dry eye symptoms and tear functions after electrocauterization treatment.

In Group 2, the mean TMA and TMH values increased after treatment but the differences were not significant (p=0.05 and p=0.85). CCA was 0.34±0.08 mm^2^ before treatment and 0.32±0.08 mm^2^ after treatment (p=0.15). The mean OSDI score significantly decreased after treatment, while TBUT did not significantly change after treatment (p<0.001 and p=0.07).

## Discussion

CCh is a senile, usually bilateral process related to conjunctival laxity in which the excess conjunctiva interposes between the globe and the lower eyelid margin.^3^ CCh localization can be temporal, nasal, central or along the entire eyelid.^3^ The presence of these folds in the conjunctiva causes a destabilization in the tear film and tear meniscus and an impairment in the tear drainage mechanism, resulting in foreign body sensation and tearing. As the vessels below the folds are fragile, minimal trauma may cause subconjunctival haemorrhage.^1,18,19^

Recent research on CCh has focused on grading, etiopathogenesis and new treatment modalities of this disorder. The most commonly used grading method in CCh is the LIPCOF grading scale.^15^ Lid-parallel conjunctival folds are evaluated in the area perpendicular to the temporal and nasal limbus above the lower lid with a slit-lamp biomicroscope and classified using the optimized grading scale. Another grading method was described by Meller and Tseng^3^ according to CCh localization, the relationship between conjunctival folds and tear meniscus, punctal occlusion, and changes with down-gaze and digital pressure.

Although the exact etiopathogenetic mechanism of CCh remains unclear, the most likely local factors are repeated conjunctival trauma, exposure to ultraviolet radiation, aging, and tear stasis. CCh accompanied by Ehlers-Danlos syndrome in a 55-year-old man showed that systemic etiologies like collagen tissue diseases must also be kept in mind in the etiopathogenesis.^20^ Current immunohistochemical studies revealed that inflammatory mediators like interleukin-1, matrix metalloproteinase (MMP)-3, MMP-9, and tumor necrosis factor-α are higher in CCh than in normal conjunctival tissue.^4,21,22^ Therefore, ocular surface inflammation has an important role in the etiopathogenesis of CCh.^2,22^ Topical anti-inflammatory therapies in clinical practice are frequently preferred in CCh treatment with artificial tears. However, patient cooperation generally decreases in long-term medical treatments, and symptoms may reappear when the topical treatment is stopped.

In the management of CCh, if medical treatment fails, surgical treatment is advised.^5^ The most preferred surgical technique is crescent-shaped excision of the CCh tissue and primary suturation of the conjunctiva.^5^ Despite its advantages, surgical treatment of CCh has some disadvantages. Conjunctival excision which is excessive and extending to inferior fornix can cause corneal problems and impaired ocular movements.^19^ Suture material can cause foreign body sensation, giant papillary conjunctivitis, granuloma, or abscess formation.^9^ These complications have led clinicians to invent new treatment modalities. In recent years electrocauterization has been preferable for clinicians as it can be applied easily and quickly in clinical practice. Local inflammation produced by the heat during cauterization promotes fixation of the loose conjunctiva to the underlying Tenon’s capsule.^9^ High-temperature electrocauteries can cause conjunctival epithelial injury, fibrosis, scar formation in Tenon’s capsule, and fornix shortening.^10^ Low-temperature cauteries may be preferable because they minimize heat spreading and cause less cell injury. Therefore, pain and scar formation are reduced while the speed of wound healing increases.^23,24,25^

In this study we compared the efficacy of electrocauterization and conventional medical drop treatment in CCh patients. We made TMA, TMH, and CCA measurements with AS-OCT using a standard measurement protocol that was developed previously for objective efficacy evaluation.^8^ Four weeks after the electrocauterization, CCA decreased while TMA and TMH increased. In Group 2, TMA, TMH, and CCA did not significantly change 4 weeks after the conventional medical treatment. We also compared the effects of electrocauterization and conventional medical drops on dry eye symptoms and signs. We used the OSDI questionnaire to evaluate dry eye symptoms and TBUT test to evaluate tear functions. Ocular surface disease index score significantly decreased after treatment in both of the groups but this decrease was more evident in the electrocauterization group. TBUT increased in only the electrocauterization group after treatment. Therefore, tear functions improved after treatment in the electrocauterization group while there was no change in the medical drop group.

AS-OCT is a useful and reproducible instrument to provide cross-sectional images of the cornea, conjunctiva, tear meniscus, and anterior chamber angle.^26,27,28^ The importance of AS-OCT in the diagnosis and follow-up of CCh was first described by Gumus et al.^8^ They obtained AS-OCT measurements before and after electrocauterization in 12 eyes of 7 patients with CCh to evaluate the reproducibility and repeatability of AS-OCT in CCh, and concluded that CCA measurement was more reliable than TMA and TMH measurements when assessing CCh with AS-OCT.^8^ The lower reliability of TMA and TMH may be due to destruction of the smooth tear meniscus in repeated measurements. The reproducibility and repeatability of AS-OCT were found to be high in measuring cross-sectional CCA, and AS-OCT was described as a useful clinical research tool for the objective study of CCh.^8^

The relationship between dry eye and CCh has been discussed many times in the literature, and CCh was found to be an important factor in dry eye etiopathogenesis.^3,29,30,31^ Loose conjunctival tissue excision alleviates dry eye symptoms and signs. In one study, crescent-shaped excision of loose conjunctival tissue and subconjunctival cauterization reduced dry eye symptoms and signs assessed with OSDI questionnaire, Schirmer tear test, and TBUT test.^32^ Another study comparing the results of electrocauterization and surgical excision in CCh treatment demonstrated significant improvement in dry eye symptoms (decrease in OSDI scores) in the electrocauterization group.^9^

In the literature, increased meibomian gland dysfunction has been reported in CCh. However, it is not known whether or how meibomian gland dysfunction is related to CCh.^3,29,30,31^ Meibomian gland function can be evaluated using meibomian gland expression, lipid layer thickness, and, indirectly, TBUT test. In the present study, TBUT values increased after electrocoagulation. Conjunctival folds at the eyelid margin may obstruct meibomian gland orifices and block meibum delivery to the tear film. As meibum aids in reducing the evaporation of tears, this obstruction may lead to a dysfunctional tear film and subsequent evaporative dry eye. Decreasing or eliminating the conjunctival folds by electrocoagulation might reopen the meibomian gland orifices, resulting in improved TBUT results.

### Study Limitations

There are some limitations in this study. The AS-OCT measurements were obtained manually from the inferotemporal wedge region between the bulbar conjunctiva and eyelid margin because we hypothesized that taking the measurements from the electrocauterized area would be more appropriate. Another limitation is that aqueous deficient dry eye was not investigated in our study.

To the best of our knowledge, there are no previous studies comparing the efficacy of electrocauterization and conventional medical drops in the treatment of CCh. Medical treatment is the most preferred treatment, although cauterization is gaining popularity in CCh treatment. Electrocauterization may be more successful than medical treatment in CCh treatment.

## Conclusion

Conjunctival cauterization is an effective and safe treatment modality in CCh. It could be a first-line treatment for CCh, especially in patients who may not want to use or may not adhere to long-term topical treatment. Further studies with larger samples are needed to evaluate the long-term effects of electrocauterization in the treatment of CCh.

## Figures and Tables

**Table 1:**
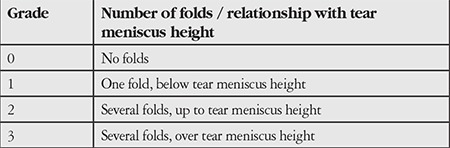
LIPCOF grading^15^

**Table 2:**
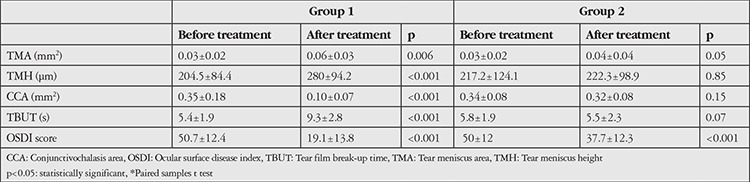
The data classified by groups

## References

[1] Hughes WL (1942). Conjunctivochalasis. Am J Ophthalmol..

[2] Ward SK, Wakamatsu TH, Dogru M, Ibrahim OM, Kaido M, Ogawa Y, Matsumoto Y, Igarashi A, Ishida R, Shimazaki J, Schnider C, Negishi K, Katakami C, Tsubota K (2010). The role of oxidative stress and inflammation in conjunctivochalasis. Invest Ophthalmol Vis Sci..

[3] Meller D, Tseng SC (1998). Conjunctivochalasis: literature review and possible pathophysiology. Surv Ophthalmol..

[4] Meller D, Li DQ, Tseng SC (2000). Regulation of collagenase, stromelysin, and gelatinase B in human conjunctival and conjunctivochalasis ﬁbroblasts by interleukin-1beta and tumor necrosis factor-alpha. Invest Ophthalmol Vis Sci..

[5] Meller D, Maskin SL, Pires RT, Tsenq SC (2000). Amniotic Membrane Transplantation for Symptomatic conjunctivochalasis refractory to medical treatments. Cornea..

[6] Kheirkhah A, Casas V, Blanco G, Li W, Hayashida Y, Chen YT, Tseng SC (2017). Amniotic membrane transplantation with fibrin glue for conjunctivochalasis. Am J Ophthalmol..

[7] Otaka I, Kyu N (2000). A new surgical technique for management of conjunctivochalasis. Am J Ophthalmol..

[8] Gumus K, Crockett CH, Pflugfelder SC (2010). Anterior segment optical coherence tomography: a diagnostic instrument for conjunctivochalasis. Am J Ophthalmol..

[9] Zhang XR, Zhang ZY, Hoffman MR (2012). Electrocoagulative surgical procedure for treatment of conjunctivochalasis. Int Surg..

[10] Kim KH, Ko AY, Ryu JS, Kim MK, Wee WR (2013). Effect of electrocauterization on the inflammation of the conjunctiva in experimental animal model. Korean J Ophthalmol..

[11] Qiu X, Gong L, Sun X, Jin H (2011). Age-related variations of human tear meniscus and diagnosis of dry eye with Fourier-domain anterior segment optical coherence tomography. Cornea..

[12] Ibrahim OM, Dogru M, Kojima T, Matsumoto Y, Wakamatsu TH, Tsubota K, Fujishima H (2012). OCT assessment of tear meniscus after punctal occlusion in dry eye disease. Optom Vis Sci..

[13] Bayhan SA, Bayhan HA, Muhafız E, Can I (2013). Evaluation of the Correlation Between Tear Meniscus Parameters and Conventional Dry Eye Tests. Turk J Ophthalmol..

[14] Demirok GS, Gurdal C, Sarac O, Ceran BB, Can I (2013). Evaluating of Tear Meniscus Parameters with Optical Coherent Tomography in Dry-Eye Patients. Turk J Ophthalmol..

[15] Höh H, Schirra F, Knienecker C, Ruprecht KW (1996). Lid-parallel conjunctival fold (LIPCOF) and dry eye: a diagnostic tool for the contactologist (in German). Contactologia..

[16] Schiffman RM, Christianson MD, Jacobsen G, Hirsch JD, Reis BL (2000). Reliability and validity of the ocular surface disease index. Arch Ophthalmol..

[17] Lemp MA (1973). Breakup of the tear film. Int Ophthalmol Clin..

[18] Yamamoto M, Hirano N, Haruta Y, Ohashi Y, Araki K, Tano Y (1994). Bulbar conjunctival laxness and idiopathic subconjunctival hemorrhage. Atarashii Ganka..

[19] Liu D (1986). Conjunctivochalasis: a cause of tearing and its management. Ophthal Plast Reconstr Surg..

[20] Whitaker JK, Alexander P, Chau DY, Tint NL (2012). Severe conjunctivochalasis in association with classic type Ehlers-Danlos syndrome. BMC Ophthalmol.

[21] Acera A, Vecino E, Duran JA (2013). Tear MMP-9 levels as a marker of ocular surface inflammation in conjunctivochalasis. Invest Ophthalmol Vis Sci..

[22] Li DQ, Meller D, Liu Y, Tseng SC (2000). Overexpression of MMP-1 and MMP-3 by cultured conjunctivochalasis fibroblasts. Invest Ophthalmol Vis Sci..

[23] Youm DJ, Kim JM, Choi CY (2010). Simple surgical approach with high-frequency radio-wave electrosurgery for conjunctivochalasis. Ophthalmology..

[24] Huang SK (1991). Advances in applications of radiofrequency current to catheter ablation therapy. Pacing Clin Electrophysiol..

[25] Hurwitz JJ, Johnson D, Howarth D, Molgat YM (1993). Experimental treatment of eyelashes with high-frequency radio wave electrosurgery. Can J Ophthalmol..

[26] Huang D, Swanson EA, Lin CP, Schuman JS, Stinson WG, Chang W, Hee MR, Flotte T, Gregory K, Puliafito CA, Fujimoto JG (1991). Optical coherence tomography. Science..

[27] Sakata LM, Lavanya R, Friedman DS, Aung HT, Gao H, Kumar RS, Foster PJ, Aung T (2008). Comparison of gonioscopy and anterior segment ocular coherence tomography in detecting angle closure in different quadrants of the anterior chamber angle. Ophthalmology..

[28] Ciancaglini M, Carpineto P, Agnifili L, Nubile M, Lanzini M, Fasanella V, Mastropasqua L (2008). Filtering bleb functionality: a clinical, anterior segment optical coherence tomography and in vivo confocal microscopy study. J Glaucoma..

[29] Wang Y, Dogru M, Matsumoto Y, Ward SK, Ayako I, Hu Y, Okada N, Ogawa Y, Shimazaki J, Tsubota K (2007). The impact of nasal conjunctivochalasis on tear functions and ocular surface findings. Am J Ophthalmol..

[30] Di Pascuale MA, Espana EM, Kawakita T, Tsenq SC (2004). Clinical characteristics of conjunctivochalasis with or without aqueous tear deficiency. Br J Ophthalmol..

[31] Uchino M, Dogru M, Yagi Y, Goto E, Tomita M, Kon T, Saiki M, Matsumoto Y, Uchino Y, Yokoi N, Kinoshita S, Tsubota K (2006). The features of dry eye disease in a Japanese elderly population. Optom Vis Sci..

[32] Wang S, Ke M, Cai X, Chen X, Yu A, Dai H, Wen X (2012). An improved surgical method to correct conjunctivochalasis: conjunctival semiperitomy based on corneal limbus with subconjunctival cauterization. Can J Ophthalmol..

